# 77. Targeting Adenosine A2B Receptor to Prevent Post-Clostridioides difficile Infection-associated Gastrointestinal Dysfunction

**DOI:** 10.1093/ofid/ofaf695.028

**Published:** 2026-01-11

**Authors:** Deiziane Costa, Maria L G Morais, Ashley K Nguyen, Joel Linden, Brian D Gulbransen, Cirle A Warren

**Affiliations:** University of Virginia, Charlottesville, VA; University of Virginia, Charlottesville, VA; University of Virginia, Charlottesville, VA; University of Virginia, Charlottesville, VA; Michigan State University, East Lansing, Michigan; University of Virginia, Charlottesville, VA

## Abstract

**Background:**

*Clostridioides difficile* (*C. difficile)* infection (CDI) is the leading cause of antibiotic-associated diarrhea worldwide. Post-CDI-associated gastrointestinal (GI) dysfunction has been reported in up to 30% of infected patients, suggesting that persistent colonic damage impacts gut function. Recently, we discovered that in the mouse model *C. difficile* induced post-infection constipation which is proportional to the intensity of intestinal inflammation at the peak of infection. Adenosine A2B receptor activation during CDI generates intense proinflammatory response. Here, we validated the clinical relevance of A2B in CDI by using human colonic biopsies and used a novel highly specific A2B antagonist, ADO5030, in combination with conditional ablation tool in the mouse model to interrogate whether A2B modulation can prevent GI dysfunction post-CDI.

**Methods:**

Colonic tissues from patients with active CDI and uninfected patients (age and gender matched) were assayed for A2B expression by immunohistochemistry. 2-3-month-old mice (littermates and conditional knockout with specific ablation of A2B in epithelial cells and in whole body cells) were infected with *C. difficile*, monitored daily and euthanized on day 7 and day 21 post-infection (pi) to assess gut inflammation and GI motility function.

**Results:**

Immunohistochemistry analysis revealed increased A2B expression in both the mucosa-including intestinal epithelial cells- and muscular colonic layers of infected patients compared to uninfected controls. Oral administration of ADO5030 accelerated recovery from CDI-associated clinical symptoms, reduced peak of infection-related gut inflammation, and significantly improved survival in mice. *In vivo*, A2B blockade effectively prevented CDI-induced delays in GI motility at day 21 pi. Furthermore, global A2B deletion, but not intestinal epithelial-specific A2B ablation, replicated the therapeutic effects of A2B antagonist treatment, suggesting that non-epithelial A2B signaling mediates these benefits.

**Conclusion:**

Our findings identify A2B as a potential clinically relevant target in patients with CDI. Our preclinical data further show that A2B modulation-independent of epithelial A2B expression, can prevent post-CDI intestinal motility dysfunction.

**Disclosures:**

Cirle A. Warren, MD, Adovate, LLC: Grant/Research Support|Ferring Pharmaceuticals, Inc.: Site PI for ROAR

